# LASSO Model Better Predicted the Prognosis of DLBCL than Random Forest Model: A Retrospective Multicenter Analysis of HHLWG

**DOI:** 10.1155/2022/1618272

**Published:** 2022-09-16

**Authors:** Ziyuan Shen, Shuo Zhang, Yaxue Jiao, Yuye Shi, Hao Zhang, Fei Wang, Ling Wang, Taigang Zhu, Yuqing Miao, Wei Sang, Guoqi Cai, Working Group Huaihai Lymphoma

**Affiliations:** ^1^Department of Epidemiology and Biostatistics, School of Public Health, Anhui Medical University, Hefei, Anhui 230032, China; ^2^Department of Hematology, Affiliated Hospital of Xuzhou Medical University, Xuzhou, Jiangsu 221006, China; ^3^Department of Hematology, The First People's Hospital of Huai'an, Huai'an, Jiangsu 223300, China; ^4^Department of Hematology, The Affiliated Hospital of Jining Medical University, Jining, Shandong 272000, China; ^5^Department of Hematology, The First People's Hospital of Changzhou, Changzhou, China; ^6^Department of Hematology, Tai'an Central Hospital, Tai'an, Shandong 271000, China; ^7^Department of Hematology, The General Hospital of Wanbei Coal-Electric Group, Suzhou, Anhui 234011, China; ^8^Department of Hematology, Yancheng First People's Hospital, Yancheng, Jiangsu 224001, China

## Abstract

**Background:**

Diffuse large B-cell lymphoma (DLBCL) is a heterogeneous non-Hodgkin's lymphoma with great clinical challenge. Machine learning (ML) has attracted substantial attention in diagnosis, prognosis, and treatment of diseases. This study is aimed at exploring the prognostic factors of DLBCL by ML.

**Methods:**

In total, 1211 DLBCL patients were retrieved from Huaihai Lymphoma Working Group (HHLWG). The least absolute shrinkage and selection operator (LASSO) and random forest algorithm were used to identify prognostic factors for the overall survival (OS) rate of DLBCL among twenty-five variables. Receiver operating characteristic (ROC) curve and decision curve analysis (DCA) were utilized to compare the predictive performance and clinical effectiveness of the two models, respectively.

**Results:**

The median follow-up time was 43.4 months, and the 5-year OS was 58.5%. The LASSO model achieved an Area under the curve (AUC) of 75.8% for the prognosis of DLBCL, which was higher than that of the random forest model (AUC: 71.6%). DCA analysis also revealed that the LASSO model could augment net benefits and exhibited a wider range of threshold probabilities by risk stratification than the random forest model. In addition, multivariable analysis demonstrated that age, white blood cell count, hemoglobin, central nervous system involvement, gender, and Ann Arbor stage were independent prognostic factors for DLBCL. The LASSO model showed better discrimination of outcomes compared with the IPI and NCCN-IPI models and identified three groups of patients: low risk, high-intermediate risk, and high risk.

**Conclusions:**

The prognostic model of DLBCL based on the LASSO regression was more accurate than the random forest, IPI, and NCCN-IPI models.

## 1. Introduction

Diffuse large B-cell lymphoma (DLBCL) is the most common histological subtype of non-Hodgkin's lymphoma, manifesting highly heterogeneity in genetic and phenotypic characteristics. The International Prognostic Index (IPI) and enhanced International Prognostic Index (NCCN-IPI) are widely used prognostic models mainly based on clinical variables such as age, stage of disease, and performance status, which are challenged due to improved treatment options, pathobiology, and life expectancy of patients with DLBCL [1–3]. Another potential reason for the limited ability to predict patient survival could be due to the reliance on traditional statistical techniques. Several studies have investigated independent risk factors for the prognosis of DLBCL using traditional statistical methods [4–8]. However, traditional regression models are limited to analyzing and synthesizing a large number of covariables and subject to overfitting, which can result in the identification of significant predictors that lack generalizability and clinical utility [9, 10]. Methods based on common prognostic factors should be further optimized.

Machine learning (ML) is widely defined as a computational strategy and a branch of artificial intelligence (AI). It automatically determines methods and parameters to obtain an optimal solution to the problem. The learning process assumes that it simulates an aspect of human intelligence and can be used for superficial intelligent purposes [9]. ML classifiers have created new opportunities for accurate and data intensive science across multiple disciplines [11, 12]. ML approaches have been used in attempts to enhance the prediction of hard-to-predict outcomes, which can also accommodate a large number of predicted values and enhance its generalization through cross-validation [10, 13].

LASSO is a regression-based methodology permitting for a large number of covariates in the model, which introduces regularization function to punish excessive fitting on the basis of logistic regression, making it compress some regression coefficients and make the coefficients with smaller absolute values to 0, so as to automatically remove unnecessary/uninfluential covariables, and can simultaneously select variables and estimate parameters [12, 14, 15]. Wang et al. constructed an immune marker of bladder cancer (BCa) by the LASSO algorithm, which had a high predictive value in the prognosis and response to immunotherapy of BCa [16]. Random forest is an ensemble learning technique developed by Breiman [17]. It is an ensemble of classifiers or regression trees with high accuracy, which looks to model response variables from a group of covariables by generating a classification tree [18]. For many practical problems with unclear prior knowledge, nonlinear multiconstraint conditions and incomplete data, the method has a good adaptive function [19]. Wu et al. identified four immune-related genes (CD48, IL1RL, PSDM3, and RXFP3) significantly associated with overall survival of DLBCL according to random forest [20].

Few studies have explored the prognostic factors of DLBCL using ML based on clinical variables [11, 21]. Therefore, this retrospective multicenter study is aimed at exploring prognostic factors of DLBCL by the LASSO and random forest model and to compare the clinical effectiveness of the LASSO, random forest model, IPI, and NCCN-IPI models.

## 2. Materials and Methods

### 2.1. Study Design

We retrospectively collected 1211 newly diagnosed DLBCL patients from August 2008 to January 2021 from 7 medical centers of the Huaihai Lymphoma Working Group (HHLWG). Patients were randomized into a training cohort (70%, *n* = 848) and a validation cohort (30%, *n* = 363). All pathological biopsies were double blinded and reviewed by at least two pathologists. Patients included in this study were treated with rituximab-based immunochemotherapy. Exclusion criteria is as follows: (1) patients with other tumors and (2) special types of lymphoma (primary central nervous system lymphoma, primary mediastinal large B-cell lymphoma, and transformed DLBCL). Ethics approval was obtained from independent Ethics Committees of each participating center in HHLWG. This study was conducted in accordance with the declaration of Helsinki.

### 2.2. Covariates

The following data of DLBCL patients in this study were recorded at enrolment: gender, age, extranodal involvement, Eastern Cooperative Oncology Group performance status (ECOG PS), presence of bulky disease (≥7.5 cm), B symptoms, albumin (ALB), white blood cell count (WBC), hemoglobin (HB), platelets (PLT), total cholesterol (TC), lymphocyte count (LYC), red blood cell count (RBC), neutrophil count (NE), height, weight, Ann Arbor stage, cell of origin, and immunological markers (BCL-2, BCL-6, and Ki-67). GCB or non-GCB phenotypes were determined by the Hans algorithm.

### 2.3. Follow-Up and Endpoints

Follow-up was conducted by reviewing inpatient medical records and making phone calls. All patients were followed up until July 28, 2021, or until death, whichever came first. Overall survival (OS) was calculated as the interval between the time of diagnosis and death from any cause or the last follow-up. The survival status of all patients was confirmed with death records or a telephone call to the patients themselves or to the next of kin of the patient (if patient died during the follow-up).

### 2.4. Statistical Analysis

Data were presented as numbers (percentages) for categorical variables and median (interquartile range, IQR) for all continuous variables. Clinical factors between the training and validation cohorts were compared using the Chi-squared test and the Mann–Whitney *U*-test. Continuous variables were transformed into categorical variables by MaxStat analysis (titled as Maximally Selected Rank Statistics).

We utilized the “glmnet” package to fit the LASSO-cox regression and used tenfold cross-validation to select the penalty term, *λ*. Random forest regression model for random forest regression analysis was constructed based on Breiman's random forest algorithm, and the Cox proportional hazards model was used to analyze the multivariable association between prognostic factors, identified in random forest regression analysis, and the OS of DLBCL. The discrimination ability of the LASSO-cox and Random forest regression models were evaluated by the receiver operator characteristic (ROC) curve analysis and Harrell's concordance index. Area under the curves (AUCs) of different models were compared using DeLong's test. For clinical usefulness, net benefit was examined against the training and validation cohorts using the decision curve analysis (DCA). Kaplan–Meier analysis was used to estimate the survival rate of DLBCL, and the log-rank test was performed for the difference between groups. All statistical analyses were performed by R software (version 4.1.3; http://www.Rproject.org).

## 3. Result

### 3.1. Patient Characteristics

In total, 1211 newly diagnosed DLBCL patients (median age 62 [range: 10-92], 54% female) with complete data were included in the final analysis. The training cohort consisted of 848 patients, and the validation cohort consisted of 363 patients. The median follow-up time was 43.4 months and the 5-year OS was 58.5%. Mann–Whitney *U* test and Chi-squared test showed that there was no significant difference in age, gender, WBC, Ki-67, ECOG PS score, and IPI between the training cohort and the validation cohort (*P* > 0.05, Table 1). The details of patients in both cohorts are shown in Table 1.

### 3.2. Variables Selection Based on LASSO Regression

**Figure 1:**
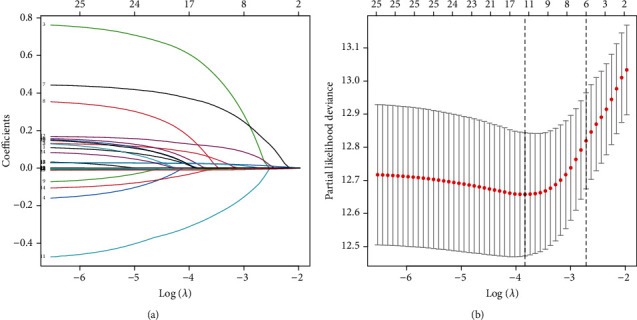
Clinical variables selection using the LASSO model.(a) The variation characteristics of variable coefficient in LASSO model; (b) the process of screening the optimum value of the parameter *λ* by cross-validation.

Figure 1(a) shows the variables with smaller coefficients (i.e., approaching zero) had a higher log Lambda. The tenfold cross-validation indicated that the optimal model could be attained at Lambda = 0.026 (Figure 1(b)). Among the 25 variables included in this study, 12 variables with the most significant correlation with the prognosis of DLBCL were screened out through the LASSO regression model. These variables were age, WBC, HB, ALB, LYC, ECOG, gender, bulky, Ann Arbor stage, spleen involvement, CNS involvement, and B symptom.

### 3.3. Random Forest Model Evaluation Index

**Figure 2:**
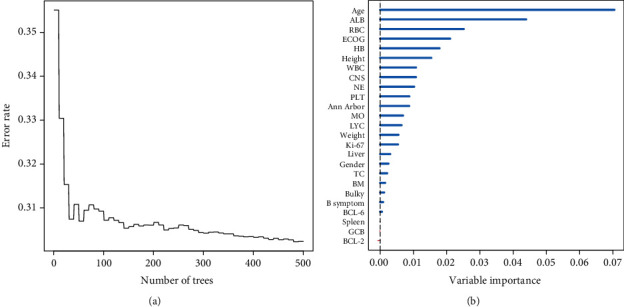
Error rate corresponding to different tree number.

In the random forest model, the error rate was relatively low and stable when the number of survival trees was 490 (Figure 2). The importance score of each predictive variable was calculated, and the features were ranked in descending order according to the importance score as follows: age, ALB, RBC, ECOG, HB, height, WBC, CNS involvement, NE, PLT, Ann Arbor stage, MO, LYC, weight, and Ki-67. Age and ALB ranked the top two positions in different datasets, which demonstrated that the two biomarkers were the important predictive variables in the DLBCL cohort.

### 3.4. The Prognostic Variables of DLBCL

To further explore the independent prognostic factors, the multivariable Cox regression analyses were carried out. The results demonstrated that age, WBC, HB, CNS involvement, gender, and Ann Arbor stage were independent prognostic factors for DLBCL on the basis of the LASSO model (*P* < 0.05). Multivariable Cox model based on random forest showed that age, WBC, HB, CNS involvement, ALB, and ECOG were indicators for the survival of DLBCL patients (Table 2).

### 3.5. Comparison of Prediction Ability between LASSO and Random Forest Model

**Figure 3:**
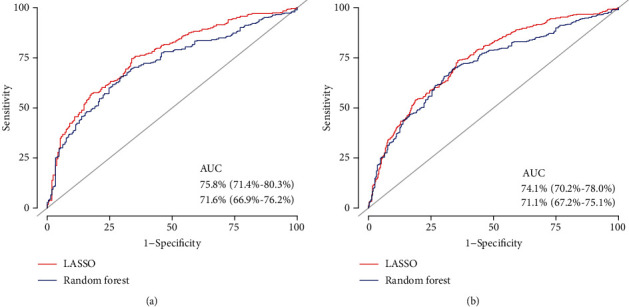
Comparison between the LASSO and random forest model of prediction ability in (a) the training cohort and (b) the validation cohort.

The LASSO model achieved an AUC of 75.8% (95% CI: 71.4%-80.3%) for predicting the prognosis of DLBCL in the training cohort, which was higher than the random forest model (AUC: 71.6%; 95% CI: 66.9%-76.2%, Figure 3(a), DeLong's test: *P* < 0.001). This result was not changed in the validation cohort (Figure 3(b)). In addition, the Harrell's concordance index was also higher for the LASSO model (LASSO: C − index = 0.704, *P* < 0.001; random forest: C − index = 0.686, *P* < 0.001).

**Figure 4:**
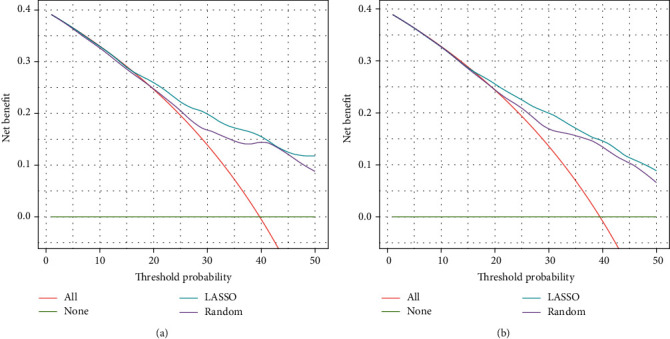
Comparison between the LASSO and random forest model of prediction ability by DCA in (a) the training cohort and (b) the validation cohort.

DCA analysis revealed that the LASSO model had higher net benefits and exhibited a wider range of threshold probabilities by risk stratification, compared to the random forest model, in predicting the prognosis of DLBCL (Figure 4).

### 3.6. Comparison of LASSO, IPI, and NCCN-IPI

**Figure 5:**
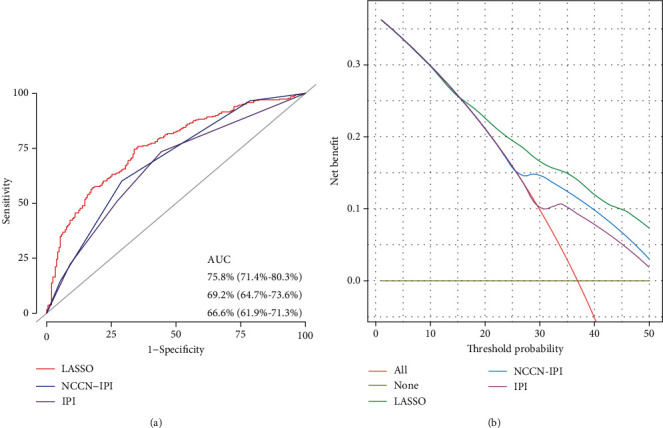
Comparison between the LASSO, IPI, and NCCN-IPI models.

All patients have complete data for the variables required to calculate the IPI and NCCN-IPI scores, and the survival curves are shown in Supplementary Figure 1. Compared with the IPI and NCCN-IPI models, the prediction accuracy of the LASSO model for DLBCL prognosis increased by 12% and 9%, respectively.  Figure 5 shows that the AUC of the LASSO model was significantly higher than both the IPI and NCCN-IPI models (DeLong's test: *P* = 0.006; *P* < 0.001). The C-index of the LASSO model was higher than that of IPI (C − index = 0.625, *P* < 0.001) and NCCN-IPI (C − index = 0.647, *P* < 0.001).

### 3.7. Stratification System Based on LASSO Model

**Figure 6:**
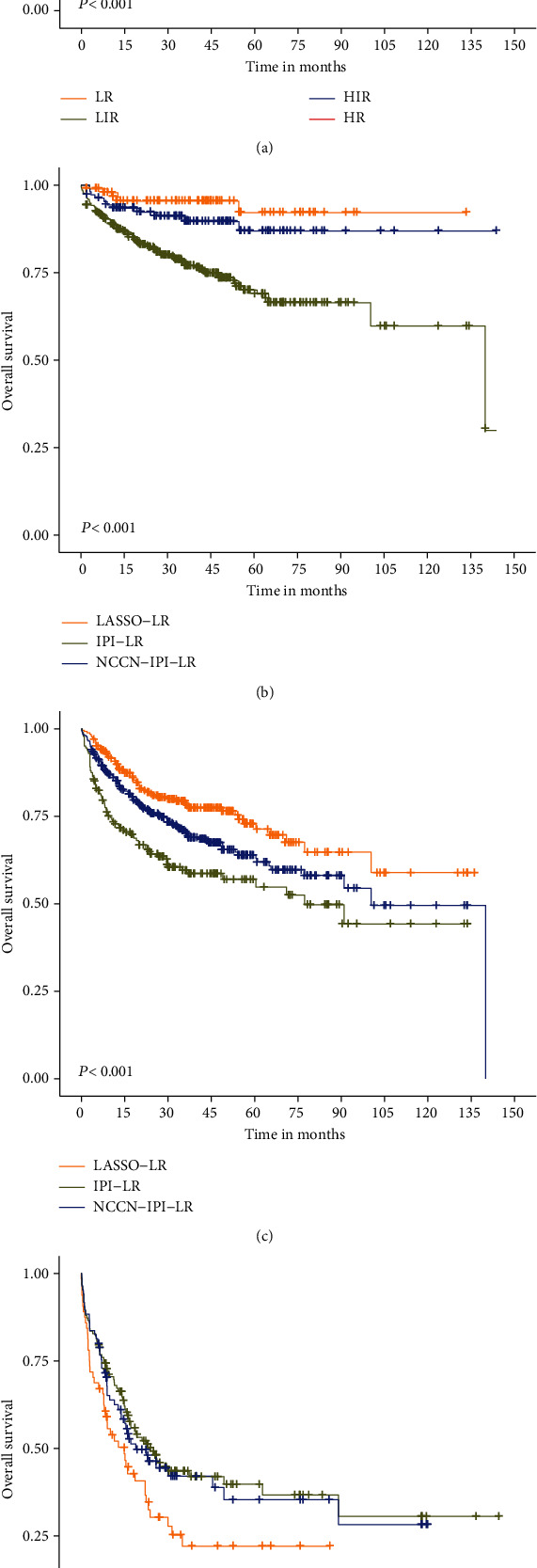
(a) Kaplan-Meier survival curves of DLBCL patients by the LASSO model; comparison of LASSO, IPI, and NCCN-IPI models in the LR (b), LIR (c), and HR groups (d).

According to the maximal Chi-squared method, 70, 104, and 8.02 were the optimal cut-off points for age, HB, and WBC, which distinguished two prognostic groups most effectively (*P* < 0.05). Based on the LASSO model, we used a maximum of 6 scoring points for categorized age (≥70), WBC (≥8.02), HB (<104), male, the presence of CNS involvement, and Ann Arbor stage III-IV, each having a score of 1. Four stratification risk groups were formed based on KM analysis: low risk (LR, 0 pt), low-intermediate risk (LIR, 1 pt), high-intermediate risk (HIR, 2-3 pts), and high-risk (HR, ≥4 pts). The LASSO model showed better discrimination of outcomes compared with the IPI and NCCN-IPI model and identified an LR group, HIR group, and HR group (Figure 6).

## 4. Discussion

In this retrospective multicenter study, we proved that the LASSO model is superior to the random forest model in predicting the prognosis of DLBCL. In addition, the model based on LASSO regression showed better discrimination of outcomes compared with the IPI and NCCN-IPI and identified a low-risk group, high-intermediate risk group, and high-risk group more precisely.

Predictive analysis is an important application of ML. For example, ML has been used to predict the prognosis of many diseases, including COVID-19, lung cancer, and stroke [22–24]. However, studies that explored the prognostic factors of DLBCL were mainly based on traditional regression models. Therefore, we built two ML models (the LASSO and random forest regression models) and identified the prognostic factors from each of them. The results suggested that the predictive performance of both sets of prognostic factors, especially factors identified from the LASSO regression model, was superior to IPI and NCCN-IPI models for the prognosis of DLBCL. This is expected given that a previous study has indicated that LASSO can enhance the prediction accuracy and interpretability of statistical models and is suitable for high-dimensional data [25]. According to LASSO regression, we found 8 new variables that may have an impact on the prognosis of DLBCL, in addition to the 4 variables included in the IPI model. Similarly, through the random forest, we also found 11 new independent variables. These new variables identified from both ML models provided further information compared to the existing prognostic models, suggesting an application of ML for predicting the prognosis of DLBCL.

Multivariable Cox proportional regression analyses using prognostic factors identified from LASSO models showed that older age, male sex, higher white blood cell level, lower hemoglobin level, and CNS involvement were risk factors of DLBCL. This is consistent with previous studies [26–30]. Female patients had a higher survival rate, which may be related to gender-associated genetic polymorphism and the mechanism of pharmacokinetics, susceptibility, and drug resistance during treatment [31]. The assessments of prediction ability, accuracy, sensitivity, and clinical utility using ROC curve, C-index, and DCA curve consistently suggested that the LASSO model was superior to the random forest model. However, we only utilized two machine learning methods and more algorithms should be adopted in future researches.

The current prognostic model was developed using LASSO regression based on clinicopathological variables and increased the accuracy to stratify the low-risk, high-intermediate risk, and high-risk groups in newly diagnosed DLBCL, compared to the IPI and NCCN-IPI models. Compared to the IPI model, the NCCN-IPI scoring model applied a refined classification of age and normalized LDH to better predict the risk of death [3]. In this study, we calculated the optimal cut-off points of age, hemoglobin, and white blood cell count by MaxStat analysis. We identified advanced age (≥70) to be associated with high risk and proved that elderly people had worse prognosis, which was consistent with previous studies [32, 33].

According to the variables screened by LASSO regression, we established a prognostic model with the highest integral at six points, and divided the patients into four risk groups. The most widely used prognostic models, IPI and NCCN-IPI, both included five clinical predictors and identified four risk groups for DLBCL by traditional regression analysis. The 5-year OS of high-risk group identified by IPI and NCCN-IPI were 39.8% and 35.3%, respectively. By contrast, the high-risk group defined by the LASSO model was 22.1%, suggesting that the LASSO model was more accurate in identifying DLBCL patients at high risks than the IPI and NCCN-IPI models. Therefore, clinical applications of the LASSO model may improve the prognosis of DLBCL patients.

In summary, in this retrospective study of real-world data, we found that LASSO model was superior to random forest in predicting the prognosis of newly diagnosed DLBCL, although both were superior to the IPI and NCCN-IPI models. More importantly, the prognosis model based on LASSO was more accurate in identifying low-risk, low-intermediate risk, and high-risk patients than the IPI and NCCN-IPI models.

## Figures and Tables

**Table 1:** The baseline characteristics between the training cohort and the validation cohort.

Variables	Training cohort	Validation cohort	*P*
	*n* = 848	*n* = 363	
Gender (%)			
Male	451 (53.2)	203 (55.9)	0.416
Female	397 (46.8)	160 (44.1)	
Age (year)	62.00 (52.00, 70.00)	62.00 (52.00, 70.00)	0.903
TC (mmol/L)	4.32 (3.68, 4.96)	4.23 (3.67, 4.96)	0.730
ALB (g/L)	38.80 (34.80, 42.80)	39.00 (34.40, 43.35)	0.744
RBC (10^12^/L)	4.10 (3.66, 4.47)	4.06 (3.73, 4.50)	0.565
HB (g/L)	124.00 (108.00, 135.00)	123.00 (108.00, 138.00)	0.442
PLT (10^9^/L)	217.00 (165.00, 269.00)	213.00 (154.00, 272.50)	0.304
LDH (U/L)	236.00 (185.00, 404.25)	233.00 (181.20, 350.00)	0.328
Ki-67	0.75 (0.60, 0.80)	0.70 (0.60, 0.80)	0.915
B symptom (%)			
Absence	636 (75.0)	264 (72.7)	0.449
Presence	212 (25.0)	99 (27.3)	
CNS involvement (%)			
Absence	761 (89.7)	333 (91.7)	0.332
Presence	87 (10.3)	30 (8.3)	
BM involvement (%)			
Absence	776 (91.5)	333 (91.7)	0.987
Presence	72 (8.5)	30 (8.3)	
Liver involvement (%)			
Absence	808 (95.3)	343 (94.5)	0.661
Presence	40 (4.7)	20 (5.5)	
Ann Arbor stage (%)			
I/II	391 (46.1)	166 (45.7)	0.954
III/IV	457 (53.9)	197 (54.3)	
NCCN-IPI (%)			
LR/LIR	477 (56.2)	193 (53.2)	0.355
HIR/HR	371 (43.8)	170 (46.8)	
IPI (%)			
LR/LIR	529 (62.4)	221 (60.9)	0.743
HIR/HR	318 (37.5)	141 (38.8)	
Bulky (%)			
Absence	799 (94.2)	343 (94.5)	0.961
Presence	49 (5.8)	20 (5.5)	

*Note*: TC: total cholesterol; ALB: albumin; RBC: red blood cell count; HB: hemoglobin; PLT: platelet; LDH: lactate dehydrogenase; CNS involvement: central nervous system involvement; BM involvement: bone marrow involvement; IPI: International Prognostic Index.

**Table 2:** Multivariable analysis of OS based on LASSO and random forest.

Variables	HR	95% CI	*P*
LASSO			
Age	1.032	1.022-1.042	<0.001
WBC	1.028	1.017-1.040	<0.001
HB	0.988	0.983-0.993	<0.001
CNS involvement	2.241	1.636-3.068	<0.001
Gender	0.659	0.524-0.829	<0.001
Ann Arbor stage	1.644	1.286-2.100	<0.001
Random forest			
Age	1.031	1.021-1.041	<0.001
WBC	1.031	1.020-1.041	<0.001
HB	0.988	0.983-0.994	<0.001
CNS involvement	1.992	1.462-2.715	<0.001
ALB	0.984	0.969-0.999	0.038
ECOG	1.295	1.011-1.659	0.040

## Data Availability

The raw data supporting the conclusions of this article will be made available by the authors, without undue reservation.

## Supplementary Materials

Supplementary Materials Supplementary Figure 1 (a) Kaplan-Meier survival curves of DLBCL patients by IPI model (b) NCCN-IPI model.
