# Supplementary figures and images for: LASSO Model Better Predicted the Prognosis of DLBCL than Random Forest Model: A Retrospective Multicenter Analysis of HHLWG
Source: J Oncol. 2022 Sep 16;2022:1618272. doi: 10.1155/2022/1618272 (PMC9507678; doi:10.1155/2022/1618272)

A

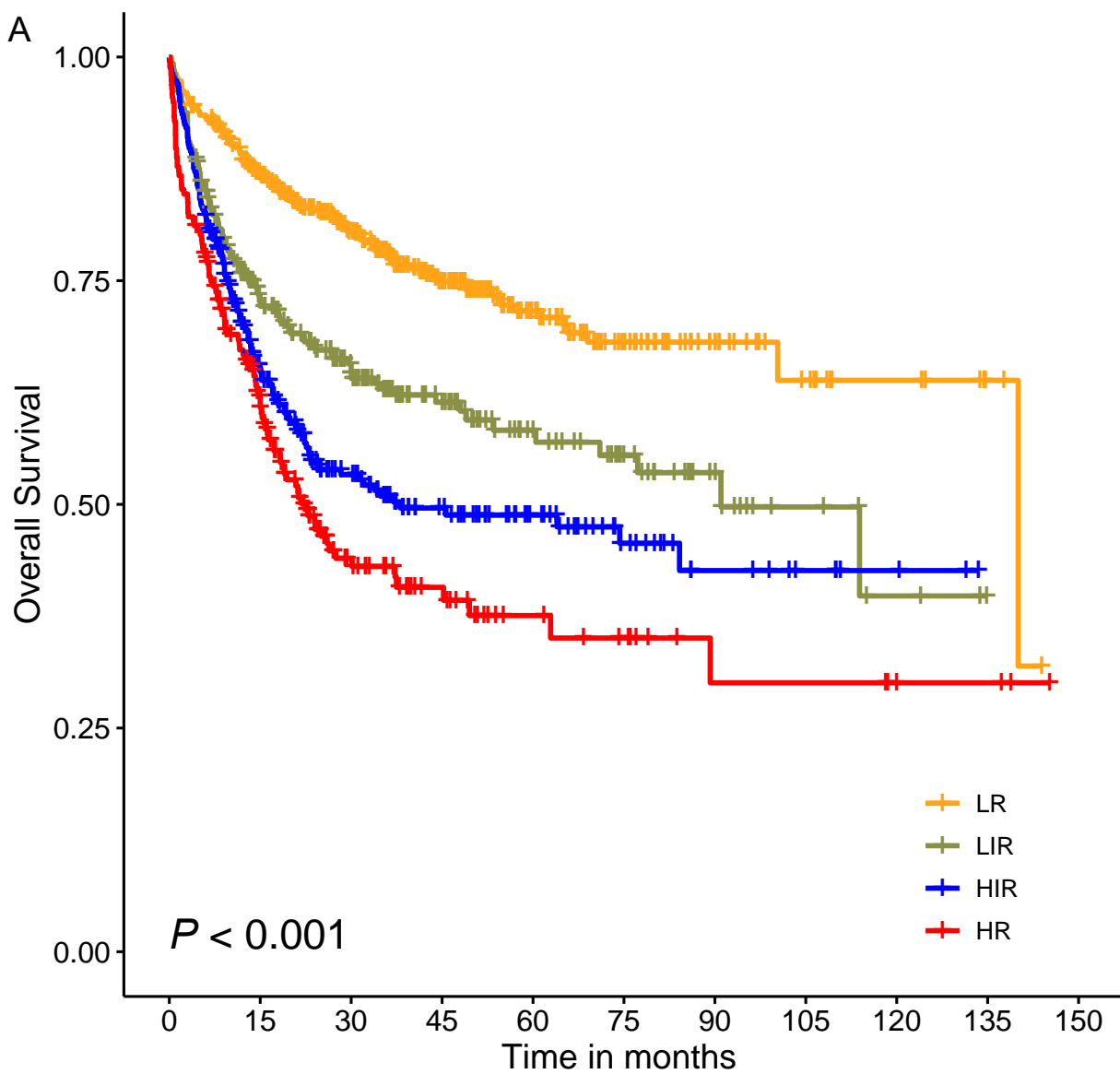

B

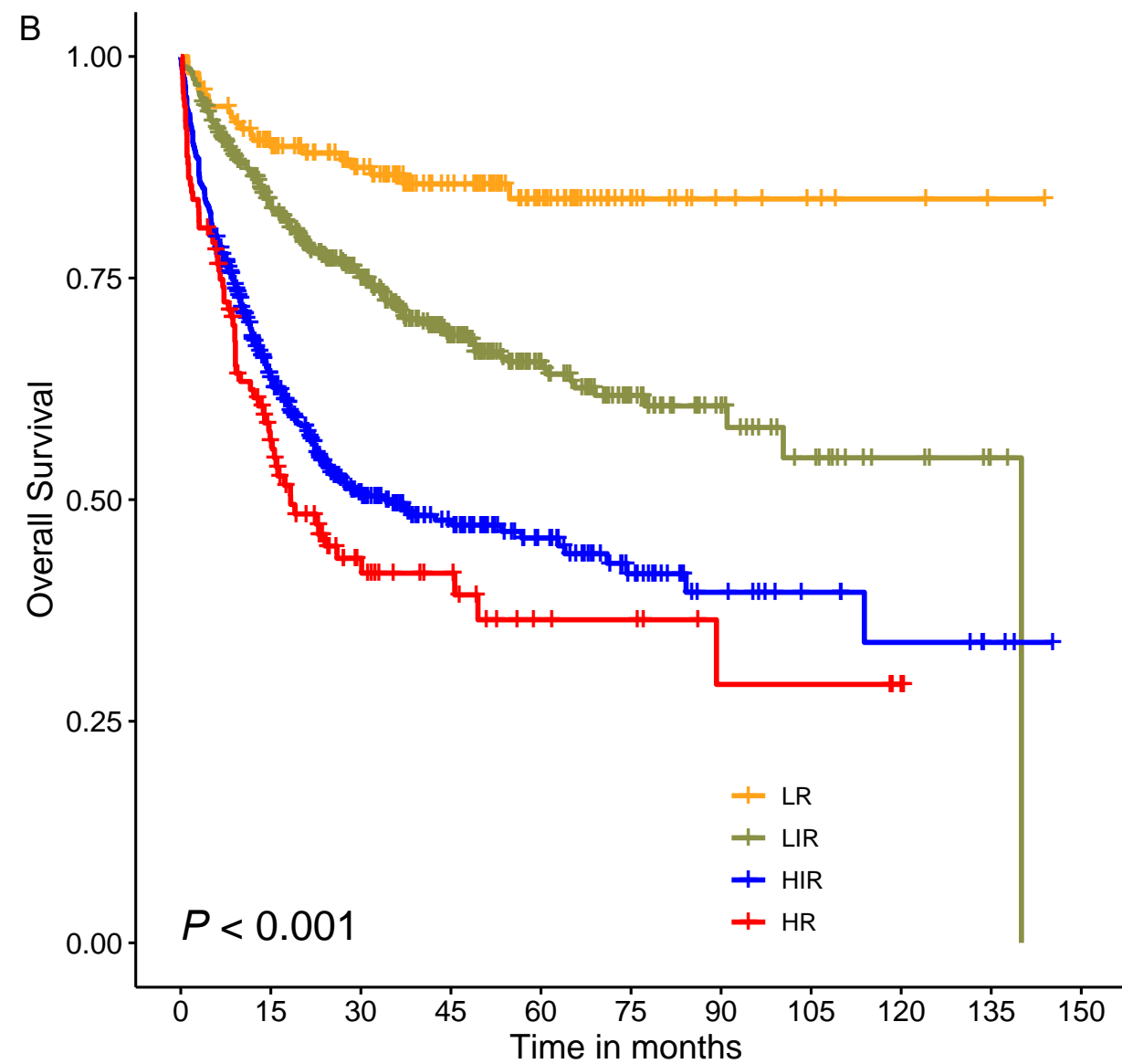

Supplement: Supplementary Materials — Supplementary Figure 1 (a) Kaplan-Meier survival curves of DLBCL patients by IPI model (b) NCCN-IPI model. [file 1618272.f1.pdf]
